# Important roles of C-terminal residues in degradation of capsid protein of classical swine fever virus

**DOI:** 10.1186/s12985-019-1238-1

**Published:** 2019-11-06

**Authors:** Yuming Chen, Erpeng Zhu, Shuangqi Fan, Hongxing Ding, Shengming Ma, Mengjiao Zhu, Shaofeng Deng, Jinding Chen, Mingqiu Zhao

**Affiliations:** College of Veterinary Medicine, South China Agricultural University, No. 483, Wushan Road; Tianhe District, Guangzhou, 510642 People’s Republic of China

**Keywords:** Classical swine fever virus (CSFV), C protein, 26S proteasome, Degradation, Cleavage

## Abstract

**Background:**

Capsid (C) protein plays an important role in the replication of classical swine fever virus (CSFV). The ubiquitin proteasome system (UPS) involves in replication of many viruses via modulation of viral proteins. The relationship of CSFV with UPS is poorly understood and the impact of 26S proteasome on C protein has never been reported before.

**Methods:**

In this study, fused C protein with an EGFP tag is expressed in PK-15 and 3D4/2 cells. MG132 and 3-methyladenine (3-MA) are used to detect the roles of 26S proteasome and autophagolysosome in expression levels of C protein. Truncated and mutant C proteins are used to find the exact residues responsible for the degradation of C protein. Immunoprecipitaion is performed to find whether C protein is ubiquitinated or not.

**Results:**

C-EGFP protein expresses in a cleaved form at a low level and is degraded by 26S proteasome which could be partly inhibited by MG132. C-terminal residues play more important roles in the degradation of C protein than N-terminal residues. Residues 260 to 267, especially M260 and L261, are crucial for the degradation. In addition, C-terminal residues 262 to 267 determine cleavage efficiency of C protein.

**Conclusions:**

CSFV C protein is degraded by 26S proteasome in a ubiquitin-independent manner. Last 8 residues at C-terminus of immature C protein play a major role in proteasomal degradation of CSFV C protein and determine the cleavage efficiency of C protein by signal peptide peptidase (SPP). Our findings provide valuable help for fully understanding degradation process of C protein and contribute to fully understanding the role of C protein in CSFV replication.

## Background

The ubiquitin proteasome system (UPS) is consist of the ubiquitin cascade reaction system and 26S proteasome. The UPS and lysosome-dependent autophagy system are two major ways of protein degradation in cells and they play significant roles in various cell activities by modulation of key protein levels [1–5]. Ubiquitination is a kind of post-translational protein modification in which ubiquitin is added to the substrate protein by covalent bonding [6]. Most proteins degraded by the 26S proteasome are modified by ubiquitination, while some are degraded in a ubiquitin-independent manner with the involvement of proteasome activator 28 (PA28) or other proteins [7, 8]. Ubiquitination mostly occurs on lysine residues and sometimes on cysteine, serine, threonine, and tyrosine residues [9]. New insights about uiquitination are being discovered with more and more researches’ focus.

Due to the close relationship of virus and host cell, many studies have shown that the UPS widely involves in virus infection and replication [10–12]. The UPS could modulate levels of viral proteins through ubiquitination and degradation [10, 11] and UPS may play complex roles during virus replication. On the one hand, degradation of viral proteins by the 26S proteasome serves as a host defense strategy to eliminate extraneous antigens [8]. On the other hand, degradation of viral proteins, such as RNA dependent RNA polymerase (RdRp), has been demonstrated to interfere with viral packaging [13, 14], may increasing viral assembly efficiency and contributing to viral evasion of the host immune system.

Classical swine fever virus (CSFV) from *Flaviviridae* family is highly pathogenic to pigs and causes great economic losses in the pig industry worldwide [15]. Its genome contains a 12.3 kb positive-sense single-stranded RNA sequence with a single large open reading frame (ORF) which encodes a polyprotein precursor that could be cleaved by cellular and viral proteases to generate 12 separate mature proteins [16, 17]. Capsid (C) protein encoding gene is located between viral gene *N*^*pro*^ and *E*^*rns*^ and is one of the four structural proteins. C protein forms by auto-catalysis of the N^pro^ at the N terminus and the cleavage of cell signal peptidase (SP) at the C terminus [18–20]. Besides, C protein is further cleaved by signal peptide peptidase (SPP) between residues A255/V256 to yield the mature C protein which contains 87 amino acids with a molecular weight (MW) about 14 kDa. SPP catalyzes intramembrane proteolysis of some signal peptides [21]. Heimann et al. found that CSFV C protein is easy to detect in concentrated virions but difficult to find in CSFV infected cells, which shows that C protein is unstable in cells [21].

CSFV C protein is important for efficient viral replication via interactions with both viral proteins and cellular proteins [22]. It has been identified that C protein interacts with viral protein NS5B and enhances its RNA dependent RNA polymerase aitivity [23]. Besides, interactions of C protein with cellular proteins SUMO-1 (small ubiquitin-like modifier 1), UBC9 (a SUMO-1 conjugating enzyme) and IQGAP1 are crucial for efficient viral proliferation and viral virulence [24, 25]. Interaction of C protein with hemoglobin subunit beta (HB) suppresses interferon-β (IFN-β) production via RNA helicases retinoic acid-inducible gene I (RIG-I) pathway by down-regulation of HB, leading to immune suppression which is beneficial for persistent CSFV replication [26].

Hepatitis C virus (HCV) is in the same family with CSFV and viral proteins core, p7, NS2 and RdRp of HCV could all be degraded by 26S proteasome, showing the close relationship of HCV and 26S proteasome [27–31]. Considering the close similarity of CSFV and HCV, the low level of C protein in CSFV infected cells, and that the relation of CSFV C protein and UPS has not been explored yet, we try to reveal the impact of UPS on CSFV C protein and explore the mechanism.

## Materials and methods

### Cells

The porcine kidney cell line PK-15 (ATCC, CCL-33) was grown in Dulbecco’s modified Eagle’s medium (DMEM) supplemented with 10% fetal bovine serum (FBS). Porcine macrophage cell line 3D4/2 (ATCC, CRL-2845) was maintained in RPMI 1640 medium (11875093, Thermo Fisher Scientific) supplemented with 10% FBS. Cells were cultured at 37 °C in a 5% CO_2_ incubator.

### Plasmids construction

**Fig. 1:**
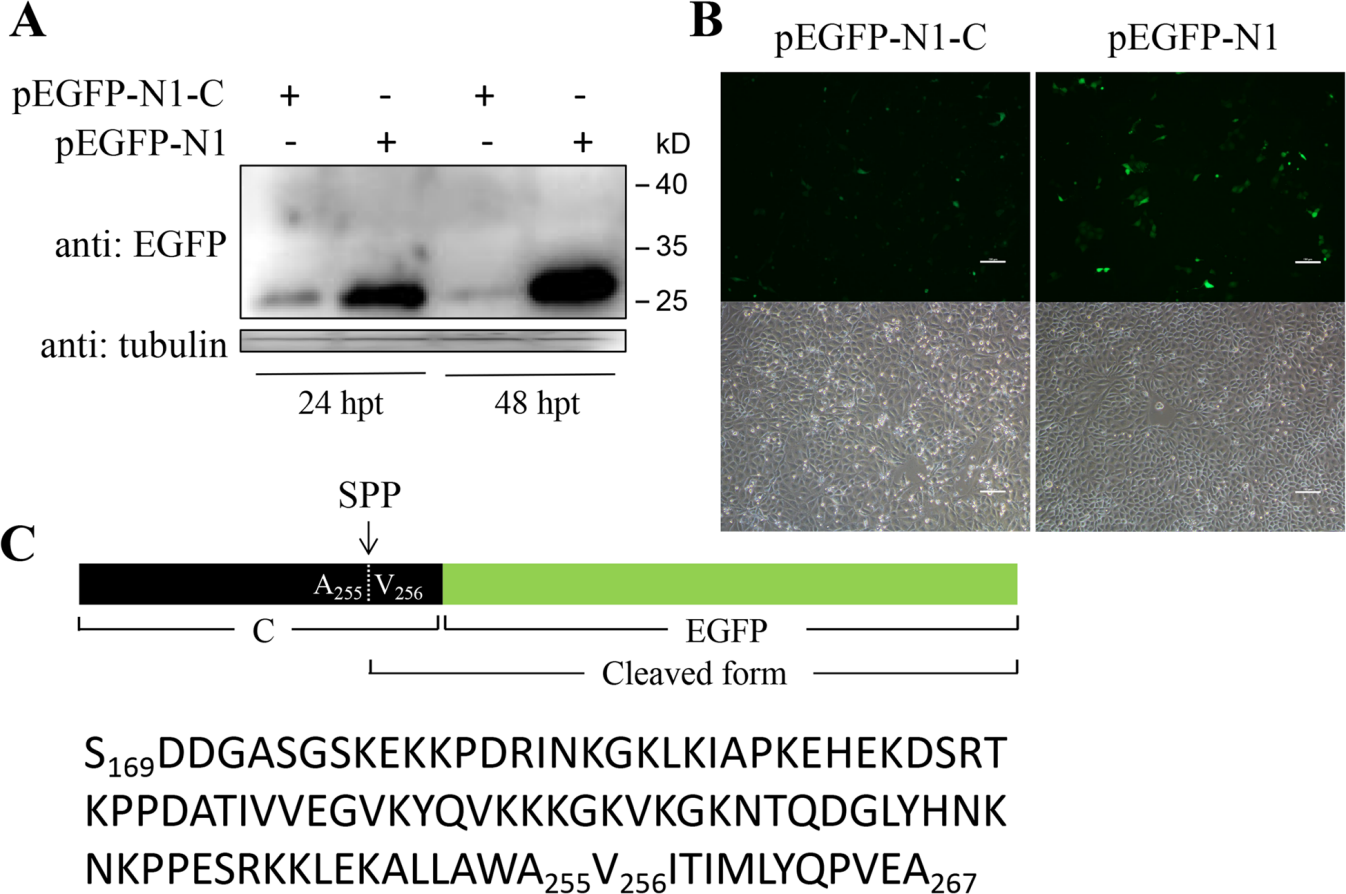
Expression of C-EGFP fusion protein in PK15 cells. **a** Plasmids pEGFP-N1-C and pEGFP-N1 were transiently transfected and cells were lysed at 24 hpt and 48 hpt. Western blot analysis was performed with mouse anti-EGFP and mouse anti-tubulin primary antibodies, and HRP-conjugated goat anti-mouse IgG secondary antibody. Tubulin was used as control. Protein marker is shown on the right. **b** PK-15 cells transfected with plasmids pEGFP-N1-C or pEGFP-N1 were observed under a fluorescence microscope at 24 hpt. Scale bar, 100 μm. **c** Schematic representation of C-EGFP protein and its cleaved form. Black frame represents C protein and green frame represents EGFP protein. The arrow indicates the cleavage site of C protein between residues A255 and V256 by SPP. Cleaved form of the fusion protein contains 12 residues at C-terminal of C protein and EGFP protein. Amino acid sequence of C protein of CSFV strain Shimen starting with S169 and ending with A267 is exhibited

**Fig. 2:**
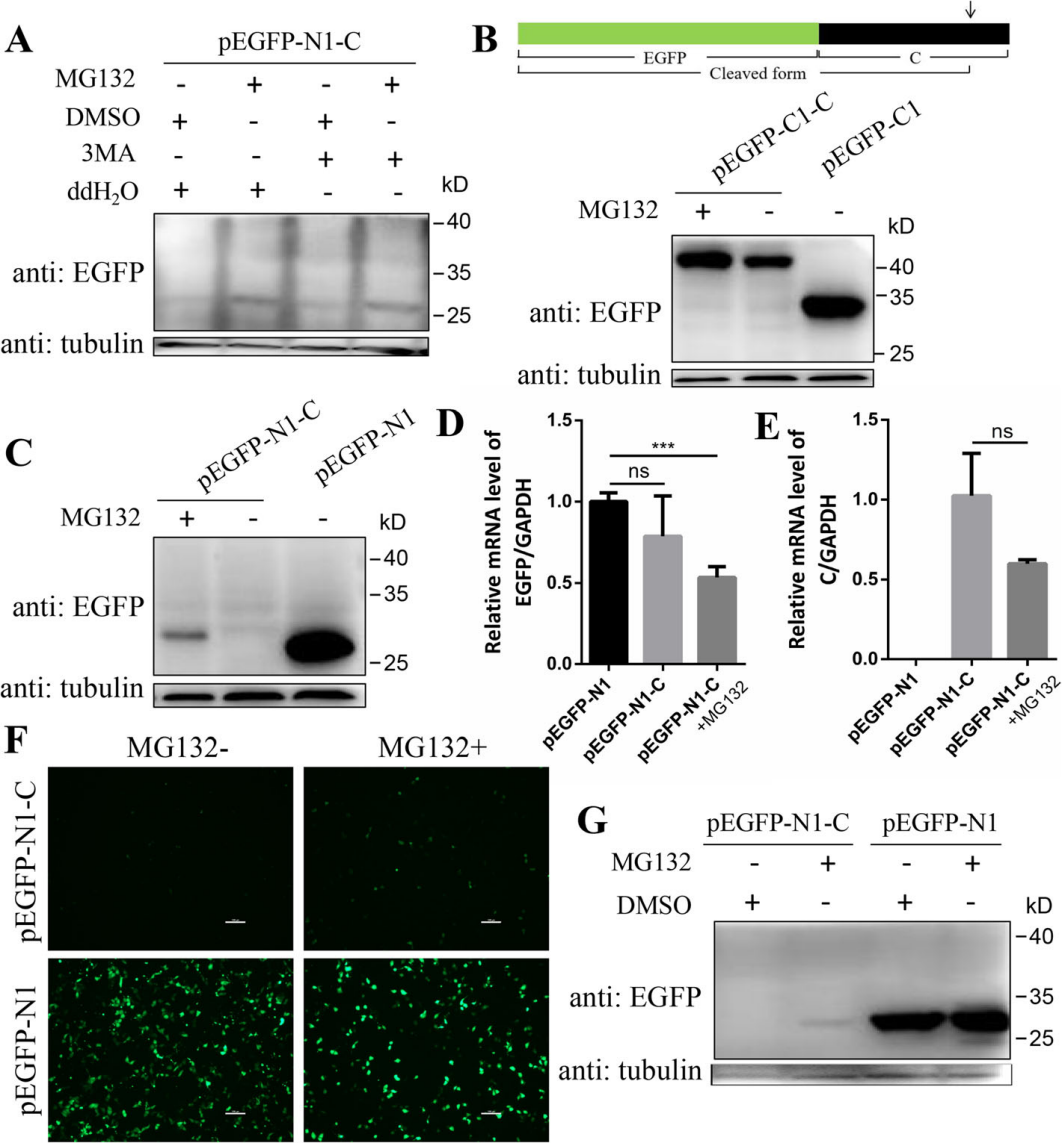
MG132 upregulated the level of EGFP-tagged C protein. **a** PK-15 cells were transfected with plasmid pEGFP-N1-C followed by treatment of 10 μM MG132 or 5 mM 3-MA or both of them for 16 h. DMSO or ddH_2_O were used as controls. At 20 hpt, cells were lysed and subjected to Western blot analysis with the indicated antibodies. Tubulin was used as a control. **b** Schematic representation of EGFP-C protein and its cleaved form. Black frame represents C protein and green frame represents EGFP protein. The arrow indicates the cleavage site of C protein by SPP. PK-15 cells were transfected with plasmid pEGFP-C1-C and were treated with or without MG132 (10 μM). Empty vector pEGFP-C1 was used as a control. Cells were lysed at 20 hpt and Western blot was performed with the indicated antibodies. **c** PK-15 cells were transfected with plasmid pEGFP-N1-C and treated with or without MG132 as described above. Empty vector pEGFP-N1 was used as a control. Western blot was performed as described above. Relative mRNA levels of EGFP (**d**) and C (**e**) in cells were detected by qRT-PCR. Data was analyzed by Student’s t-test. **f** 3D4/2 cells were transfected with plasmid pEGFP-N1-C or pEGFP-N1 followed by treatment of 10 μM MG132 or same volume of DMSO for 16 h. The fluorescence in cells was observed at 20 hpt. Scale bar, 100 μm. **g** Cells in (**f**) were then lysed and analysed by Western blot with the indicated antibodies. Protein marker is shown on the right. Data in all bar plots are shown as mean ± SD and representative of 3 biological replicates. ***, *P*< 0.001; ns, not significant

**Fig. 3:**
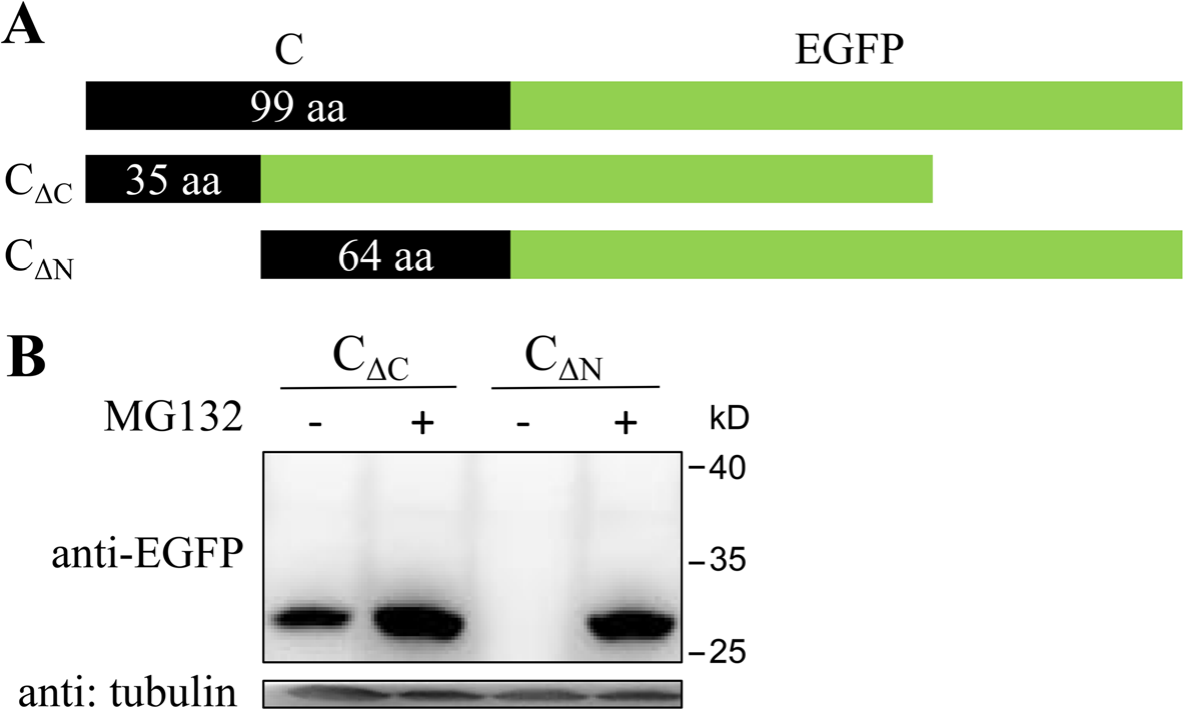
C-terminal residues are more responsible for the degradation of C protein. **a** Schematic representation of C-EGFP, C_ΔC_ and C_ΔN_ proteins. The numbers in dark oblong showed the remaining residues of C protein. C_ΔC_ is constructed by fusing the first 35 amino acids at N-termianl of C protein to EGFP protein. C_ΔN_ is constructed by fusing the last 64 amino acids at C-termianl of C protein to EGFP protein. **b** PK-15 cells were transfected with plasmids encoding C_ΔC_ and C_ΔN_ proteins followed by treatment of MG132 or DMSO as described above. Cells were lysed and analysed by Western blot with the indicated antibodies. Protein marker is shown on the right

**Fig. 4:**
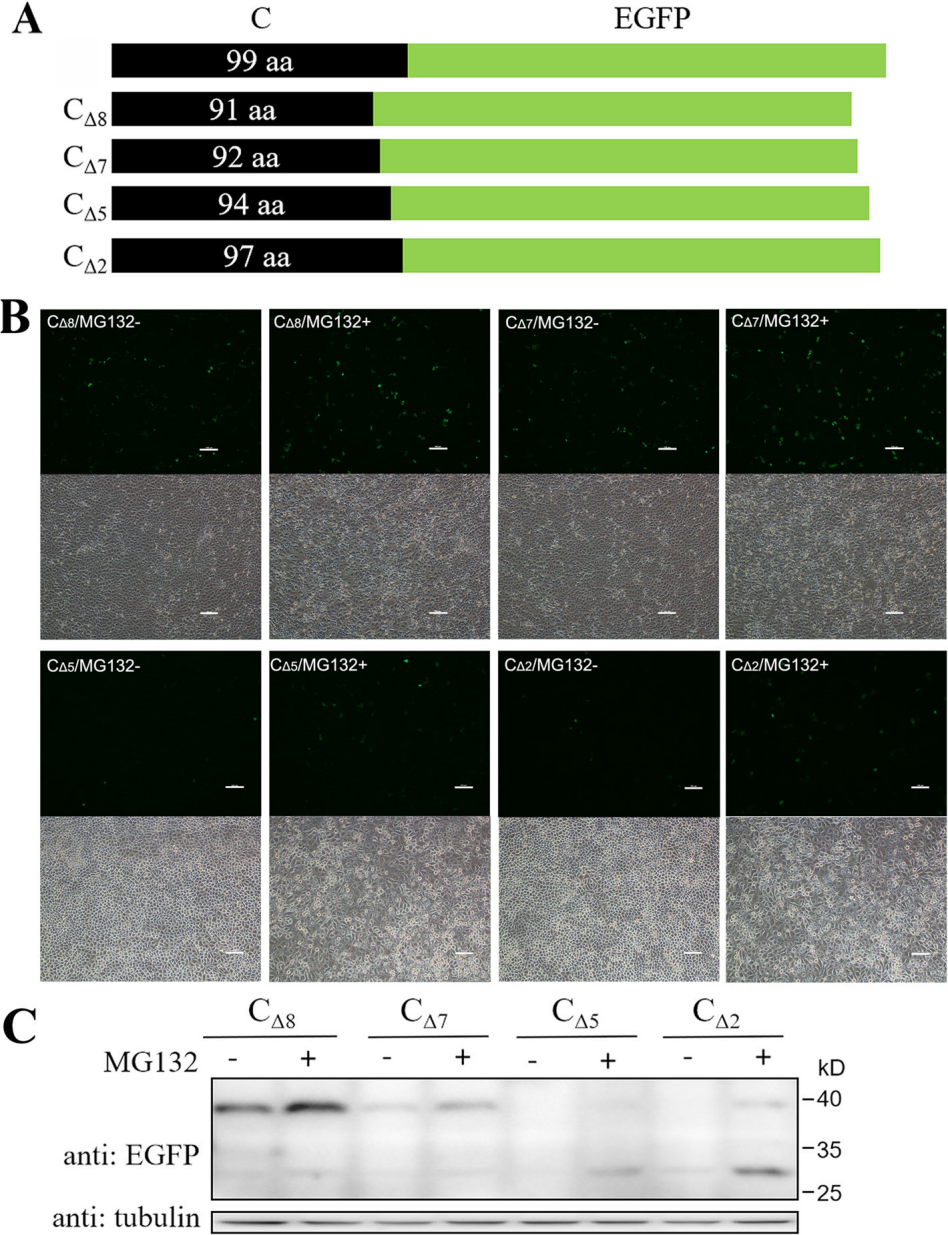
C-terminal residues 260 to 267, especially 260-262, are responsible for the degradation of C protein. **a** Schematic representation of a series of truncated fusion proteins with various residues deletions at C-terminal of C proteins. The numbers in dark oblong showed the remaining residues of C protein. **b** PK-15 cells were transfected with plasmids encoding C_Δ8_, C_Δ7_, C_Δ5_ and C_2_ proteins, respectively. Cells were treated with MG132 or DMSO for 16 h and observed under a fluorescence microscope**.** Scale bar, 100 μm. **c** Cells were then lysed and analysed by Western blot with the indicated antibodies. Protein marker is shown on the right

**Fig. 5:**
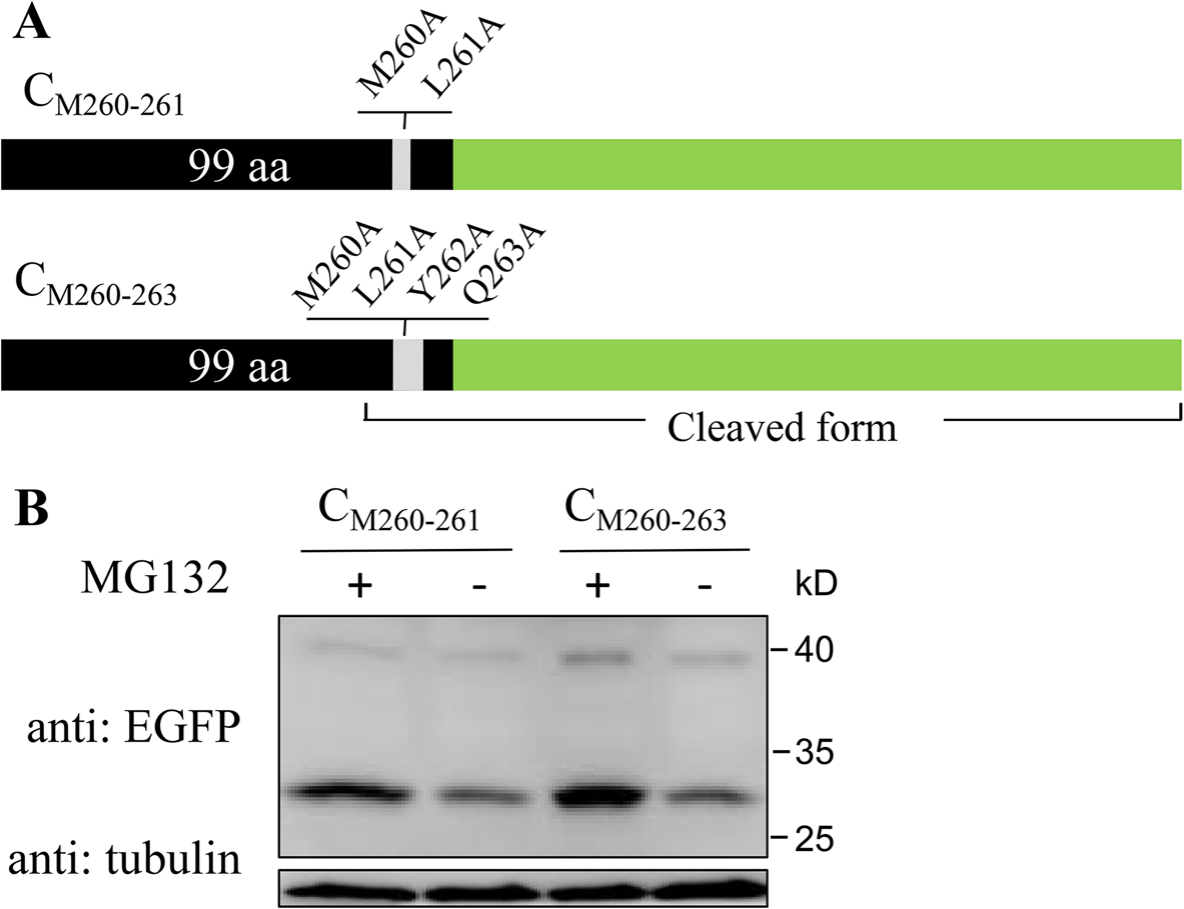
Mutations of residues M260A and L261A partly restrained C protein degradation. **a** Schematic representation of mutant C proteins, C_M260-261_ and C_M260-263_, in which residues 260-261 and 260-263 in C protein are all mutated to Alanine (A), respectively. Mutant residues are shown above the oblong. **b** C_M260-261_ and C_M260-263_ were expressed in PK-15 cells followed by treatment of MG132 or DMSO as described above. Western blot was performed and C_M260-261_ and C_M260-263_ were detected with the indicated antibodies. Protein marker is shown on the right

Plasmids pEGFP-N1-C and pEGFP-C1-C were constructed by cloning C protein-encoding gene of CSFV strain Shimen (GenBank accession no. AF092448.2) into pEGFP-N1 and pEGFP-C1 vectors (Clontech), respectively. Plasmid pEGFP-N1-C encodes C-EGFP protein fused with EGFP tag at C-terminal (Fig. 1c), and pEGFP-C1-C encodes EGFP-C protein fused with EGFP tag at N-terminal (Fig. 2b). A series of plasmids encoding truncated forms of C protein, C_ΔC_, C_ΔN_, C_Δ8_, C_Δ7_, C_Δ5_ and C_Δ2_ (Fig. 3a and 4a), with various amino acid deletions were generated from pEGFP-N1-C by conventional PCR with the primers listed in Table 1. The constructs encoding mutant forms of C protein, C_M260–261_ and C_M260–263_ (Fig. 5a), were generated by cloning the corresponding mutant sequences into pEGFP-N1 vector. All plasmids were verified by DNA sequencing to ensure that the target gene is in frame with the EGFP-coding sequence, with no intervening stop codons.

**Table 1:** Primers used in this study

Primer	Sequence (5′–3′)	Use
C-F	GTACTCGAGATGTCTGATGATGGCGCA	Amplification of C, C_ΔC_, C_Δ8_, C_Δ7_, C_Δ5_ and C_Δ2_
C_ΔN_-F	TACTCGAGATGCCACCCGACGCT	Amplification of C_ΔN_
C-R	ATGGATCCCGGGCTTCAACTGGTT	Amplification of C and C_ΔN_
C_ΔC_-R	ATGGATCCCGCTTAGTTCTGCTGTC	Amplification of C_ΔC_
C_Δ8_-R	CGGATCCCGAATTGTTATTACCGC	Amplification of C_Δ8_
C_Δ7_-R	CGGATCCCGCATAATTGTTATTAC	Amplification of C_Δ7_
C_Δ5_-R	CGGATCCCGATACAACATAATTGT	Amplification of C_Δ5_
C_Δ2_-R	CGGATCCCGAACTGGTTGATACAA	Amplification of C_Δ2_
qPCR-EGFP-F	ATGGCCGACAAGCAGAAGAA	qRT-PCR for detection of EGFP
qPCR-EGFP-R	AACTCCAGCAGGACCATGTG	
qPCR-C-F	ACAGCAGAACTAAGCCACCC	qRT-PCR for detection of C
qPCR-C-R	TCTTGTTGTGGTACAGGCCG	
qPCR-GAPDH-F	GAAGGTCGGAGTGAACGGATTT	qRT-PCR for detection of GAPDH
qPCR-GAPDH-R	TGGGTGGAATCATACTGGAACA

**Fig. 6:**
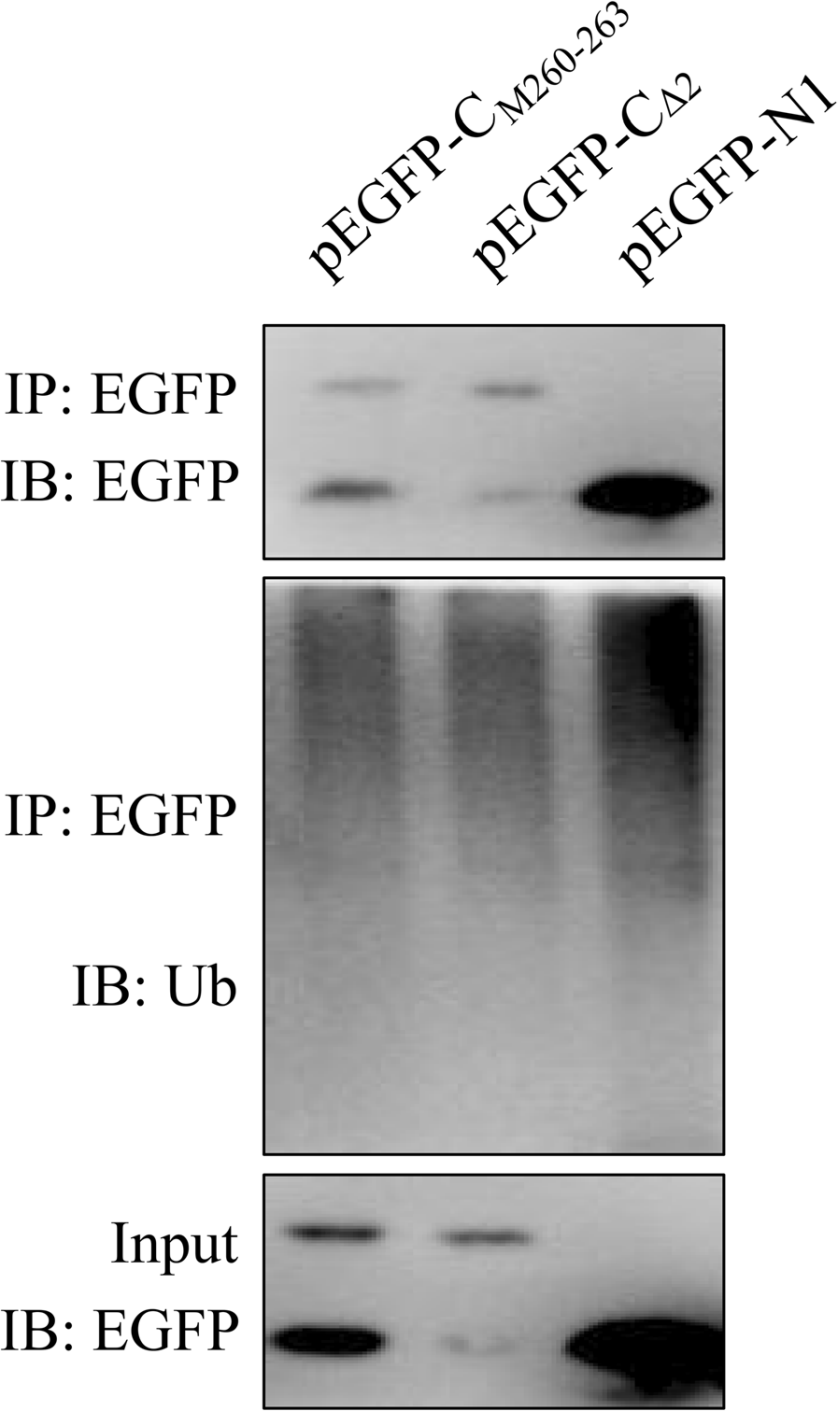
C protein is not ubiquitinated. Plasmids encoding proteins C_Δ2_, C_M260-263_ and EGFP were transiently transfected in PK-15 cells followed by treatment of MG132 (10 μM) for 16 h. Cells were lysed and immunoprecipitation was performed. Proteins in cell lysis and proteins pulled-down are detected by Western blot using anti-EGFP and anti-Ub antibodies

### Plasmids transfection

At about 70% confluence, cells in 12-well plates cultured at 37 °C in a humidified incubator with 5% CO_2_ were transfected with the indicated plasmids (1.5 μg each) using Lipo6000™ transfection reagent (C0528, Beyotime) according to the manufacturer’s instructions. At 4 h post-transfection (hpt), the transfection mixture was replaced with DMEM or drugs-containing DMEM supplemented with 2% FBS. Cells were collected at the indicated time points for the following Western blot analysis or were subjected to indirect immunofluorescence assay.

### Biochemical intervention

PK-15 or 3D4/2 cells are treated with 10 μM MG132 (A2585, Apexbio) diluted in dimethyl sulfoxide (DMSO) or 5 mM 3-methyladenine (3-MA) (A8353, Apexbio) diluted in sterile ddH_2_O for 16 h. The same amount of DMSO or ddH_2_O was added to the inoculum as controls.

### Quantitative real-time PCR

Total RNA was extracted from cells and was then reverse transcribed to cDNA with Moloney murine leukemia virus reverse transcriptase (2641A, TaKaRa) according to the manufacturer’s instructions. Gene expression was quantified by quantitative real-time polymerase chain reaction (qRT-PCR) with TB Green® *Premix Ex Taq*™ II (RR820A, TaKaRa) in the CFX96 real-time PCR system (Bio-Rad). The primers used to detect EGFP, C and GAPDH are listed in Table 1. The relative abundance of each target was obtained by normalization with endogenous GAPDH.

### Immunoprecipitation

Immunoprecipitation was performed as previously described. Briefly, PK-15 cells transfected with the indicated plasmids were lysed, and the supernatants obtained by centrifuging were incubated with anti-EGFP antibody and protein A/G agarose (P2055, Beyotime) at 4 °C overnight. The beads were then rinsed with PBS and subjected to SDS-PAGE. Protein bands were detected by Western blot using anti-EGFP and anti-Ub (sc-166,553, Santa) antibodies.

### Western blot analysis

Protein samples obtained from cell lysates were mixed with 5× protein loading buffer, boiled at 100 °C for 10 min and then subjected to SDS-PAGE. Proteins were separated and transferred to a PVDF membrane (Roche). The nonspecific antibody binding sites were blocked with 5% skim milk in PBS-Tween (PBST). Membranes were incubated with mouse anti-EGFP (AG281, Beyotime) (1: 2000) or mouse anti-tubulin (AT819, Beyotime) (1: 2000) primary antibodies diluted in PBS at 4 °C overnight, and then incubated with HRP-conjugated goat anti-mouse IgG secondary antibody diluted in PBS (1: 1000) at 37 °C for 1 h. The signals were detected using an ECL imaging system. The images were obtained from a CanoScan LiDE 100 scanner (Canon) and quantities of protein blots were measured with Image J software.

## Results

### C-EGFP fusion protein expresses in a cleaved form in PK-15 cells

To explore the expression characteristics of exogenous C protein, recombinant plasmid pEGFP-N1-C was transfected in PK-15 cells. Empty vector pEGFP-N1 was transfected as a control. Western blot analysis of samples from plasmid-transfected cells showed that no putative C-EGFP fusion protein band was detected at the expected position (27 + 14 kD) at 24 hpt and 48 hpt, while a weak band with a molecular weight (MW) close to EGFP tag (27 kD) was detected (Fig. 1a). Since C protein was previously reported to be cleaved between residues A255 and V256 by SPP, we assumed that C-EGFP fusion protein was possibly cleaved to generate an N-terminal fragment with 87 amino acids and a C-terminal fragment with 12 residues fused to the EGFP tag (termed as cleaved form below) (Fig. 1c). Therefore, the protein bands in four panels have a similar molecular weight. Besides, cleaved C-EGFP protein expressed at a very low level compared with EGFP protein and less cleaved C-EGFP protein was detected at 48 hpt than 24 hpt (Fig. 1a), showing that C protein may be degraded after expression. The fluorescence result also showed that much less fluorescent signal was observed in cells transfected with pEGFP-N1-C than pEGFP-N1 (Fig. 1b).

### C protein is degraded by 26S proteasome

Considering that C-EGFP protein expressed at a low level and decreased over time, we assume that C-EGFP protein might be degraded after expression. Since degradation of intracellular proteins are generally mediated by 26S proteasome and autophagolysosome, we added inhibitors, MG132 and 3-MA, of them to the cell medium, respectively, after plasmid transfection. MG132 is commonly used in experiments about degradation of proteins by 26S proteasome. Results showed that MG132 increased protein level of cleaved C-EGFP in PK-15 and 3-MA had no effect on protein level of cleaved C-EGFP (Fig. 2a). To examine whether the 87 residues at N-terminal were affected by 26S proteasome or not, plasmid pEGFP-C1-C encoding EGFP tagged C protein at N-terminal, EGFP-C (Fig. 2b), was constructed and transfected in PK-15 cells. EGFP-C protein could be detected and MG132 up-regulated its level (Fig. 2b), showing that the 87 residues at N-terminal may be less affected by 26S proteasome than the 12 residues at C-terminal. Plasmids pEGFP-N1-C and pEGFP-N1 were transfected in PK-15 cells, respectively. Western blot results showed that cleaved form of C-EGFP protein expressed at a relatively low level compared with EGFP protein (Fig. 2c). Transcription levels of EGFP and C were analysed by qRT-PCR and the result showed that MG132 down-regulated average mRNA level of both EGFP (Fig. 2c-d) compared with the untreated group, though the impact of MG132 on mRNA level of C was not significant. Although MG132 has opposite functions on mRNA and protein level of C protein, the conclusion that MG132 up-regulated protein level of C protein could also be obtained. Similar results were observed in 3D4/2 cells, that MG132 up-regulated the level of C-EGFP protein (Fig. 2f) and fluorescence intensity (Fig. 2g). Since protein level of EGFP was not upregulated by MG132 (Fig. 2g), we concluded that C protein was the target of 26S proteasome.

### C-terminal residues are more important for the degradation of C protein

To reveal the degradation details of C protein, two truncated C proteins with N- or C-terminal residues deletion were designed (Fig. 3a) and expressed in PK-15 cells. C_ΔC_ was detectable and MG132 up-regulated its protein level (Fig. 3b). C_ΔN_ was not detectable and MG132 up-regulated its protein level, too (Fig. 3b). These results showed that protein levels of both C_ΔC_ and C_ΔN_ were regulated by 26S proteasome. Comparing protein levels of C_ΔC_ and C_ΔN_ without MG132, it seemed that C_ΔN_ was more easily affected by 26S proteasome, showing the greater effect of C-terminal residues on the degradation of C protein.

### Determination of residues responsible for the degradation of C protein

To further confirm the exact residues critical for the degradation of C protein, a series of truncated proteins with various amino acid deletions at C-terminal of C protein were designed and expressed in PK-15 cells (Fig. 4a). Results showed that expression of C_Δ8_ protein was apparently detectable compared with C_Δ7_ (weak expression), C_Δ5_ (no expression) and C_Δ2_ (no expression) in the absence of MG132 (Fig. 4c). Therefore, the three different residues, M260, L261 and Y262, between C_Δ8_ and C_Δ5_ seemed critical for the degradation of C protein. MG132 increased the quantities of both immature and cleaved forms of C_Δ8_, C_Δ7_, C_Δ5_ and C_Δ2_ (Fig. 4c), showing that all these proteins were modulated by 26S proteasome. Besides, MG132 up-regulated fluorescence intensity in cells expressing C_Δ8_, C_Δ7_, C_Δ5_ and C_Δ2_ (Fig. 4b).

### Residue mutations of M260A and L261A partly inhibit C protein degradation

To further confirm the roles of residues M260, L261 and Y262 in the degradation of C protein, site-directed mutagenesis-based study was conducted. Two mutant proteins, C_M260–261_ in which amino acid residues 260 and 261 were mutated to Alanine (A) and C_M260–263_ in which amino acid residues 260 to 263 were mutated to Alanine (A), were designed (Fig. 5a) and expressed in cells. Results showed that both C_M260–261_ and C_M260–263_ could be detected in full length without MG132 (Fig. 5b), confirming that M260 and L261 constituted the minimum domain that was responsible for the degradation of C protein via 26S proteasome.

### CSFV C protein is not ubiquitinated

To investigate whether degradation of C protein is ubiquitin dependent, proteins C_Δ2_ and C_M260–263_ were expressed in cells with MG132. EGFP was expressed as a control. Immunoprecipitation was performed and ubiquitin was detected by Western blot. Results showed that proteins C_Δ2_, C_M260–263_ and EGFP were pulled down (Fig. 6). Unexpectedly, ubiquitin level in C_Δ2_ and C_M260–263_ panels were even lower than the EGFP panel (Fig. 6), indicating that degradation of C protein is not ubiquitin dependent.

## Discussion

PK-15 and 3D4/2 are two cell lines that have a wide variety of applications in porcine virology studies [32–34]. In this study, C-EGFP fusion protein with EGFP tag at C-terminal was not detected at the predicted position and cleaved C-EGFP protein was detected at the position with a molecular weight close to EGFP tag (Fig. 2a). EGFP-C protein fused with EGFP tag at N-terminal was detected at the predicted position (Fig. 2b). MG132 up-regulated protein levels of immature or cleaved forms of C-EGFP, EGFP-C and truncated C proteins (C_ΔC_, C_ΔN_, C_Δ8_, C_Δ7_, C_Δ5_ and C_Δ2_), showing the strong and extensive effect of 26S proteasome on the degradation of C protein. In a study about the cleavage of C protein, Heimann et al. found that CSFV C protein is difficult to detect in CSFV infected cells. Consistent with this finding, we find that small tagged C protein (HA or Flag tag) is hardly to detect in PK-15 cells, so we use the big EGFP tag in this study. And the results show that EGFP-tagged C protein could express at a low level. Taken together, the fusion protein was indeed cleaved in cells and EGFP tag prevents C protein from degrading to some extent. This may be because that C protein with a big tag covers part of the residues critical for the recognition of C protein by 26S proteasome.

Apart from degradation, we also observed changes in cleavage of C protein by SPP in expressions of truncated C proteins. Since CSFV C protein has previously been identified to be cleaved between residues A255 and V256, truncated C proteins containing this site are considered to be cleaved. In this study, C_Δ8_ and C_Δ7_ expressed mainly in uncleaved form (Fig. 4c), while C_Δ5_, C_Δ2_, C_M260–261_ and C_M260–263_ expressed mainly in cleaved form (Fig. 4c and 5b). While the SPP recognition site in C protein is from E248 to A267 [35], we conclude according to the results in this study that the last 6 residues (262 to 267) are extremely critical for the cleavage. By comparing expressions of C_Δ8_, C_Δ7_, C_Δ5_ and C_Δ2_, we further conclude that cleavage efficiency increased when there are more residues at C-terminal. Since cleavage efficiency is modulated by the last 6 residues (262 to 267) and degradation of C protein is larged determined by the last 8 residues (260 to 267), the last several residues seem to play dual functions in the process of C protein. Cleavage helps to eliminate the 26S proteasome recognition site which plays a major role in C protein degradation, increasing C protein quantity. However, since N-terminal also has partial effect on C protein degradation (Fig. 3), cleavage only partially inhibits C protein degradation. Strong degradation of viral protein by 26S proteasome may be a method employed by the host to restrict virus replication, a mechanism that has been identified in HCV, tomato bushy stunt virus (TBSV) and other viruses [8, 36, 37].

Proliferation of CSFV is widely modulated by host cellular proteins [22]. CSFV C protein has been reported to interact with the osteosarcoma amplified 9 protein (OS9) [38], a glycoprotein located in endoplasmic reticulum (ER) which plays a role in the degradation of unfolded proteins [39]. Interestingly, OS9 interacts only with the immature form of CSFV C protein and the interaction is mediated by the last 12 residues at C-terminal of C protein. Recombinant CSFV containing C protein lacking the ability to interact with OS9 showed a significantly decreased ability to replicate in cell cultures compared with the parental virus [38]. Though C protein exists in viron only in mature form and residues 179 to 180, 194 to 198, and 208 to 212 are identified significant for generation of progeny virus [40], immature C protein and its C-terminal residues seem also play important roles in CSFV replication. Since OS9 also regulates protein degradation, whether OS9 plays a role in degradation of CSFV C protein needs further investigation.

Degradation and cleavage of core protein of HCV, which is in the same faimly with CSFV, are revealed previously in several studies. Core protein of HCV could be degraded by 26S proteasome in both ubiquitin-dependent and -independent proteasomal pathways [8]. Maturation of HCV immature core protein by SPP helps to promote viral proliferation efficiency and inhibition of maturation process causes endoplasmic reticulum stress which could be restricted by TRC8-dependent proteasomal degradation of immature core protein [31]. Similar to HCV C protein, degradation and cleavage of CSFV C protein may be two independent processes that play contrary roles in the modulation of level of C protein. In the counteract between host and CSFV, 26S proteasome might be employed by the host to decrease the level of viral C protein and SPP might be utilized by CSFV to ensure enough mature C protein for replication. Considering the widely modulation roles of 26S proteasome on cell activities, the role of degradation of C protein in CSFV replication needs further detailed investigation.

## Conclusions

In this study, we identify for the first time the impact of 26S proteasome on C protein of CSFV. C protein is degraded by 26S proteasome, which could be partially inhibited by MG132. Though degradation of C protein is regulated by different parts, C-terminal residues seem more responsible than N-terminal residues. C-terminal amino acids, especially residues 262 to 267, are crucial for the cleavage of C protein by SPP. These results provide valuable reference for elucidating the mechanism underlying the degradation and maturation of CSFV C protein, and bring new insights into better understanding the biological processes of C protein of CSFV.

## Data Availability

The data supporting the findings in the current study are available from the corresponding author or the first author on reasonable request.

## Abbreviations

3-MA: 3-methyladenine
C: Capsid
CSF: Classical swine fever
CSFV: Classical swine fever virus
DMEM: Dulbecco’s modified Eagle’s medium
DMSO: Dimethyl sulfoxide
ER: Endoplasmic reticulum
FBS: Fetal bovine serum
HB: Hemoglobin subunit beta
HCV: Hepatitis C virus
hpt: hours post-transfection
IFN-β: Interferon-β
MW: Molecular weight
ORF: Open reading frame
OS9: Osteosarcoma amplified 9
PA28: Proteasome activator 28
qRT-PCR: quantitative real-time polymerase chain reaction
RdRp: RNA dependent RNA polymerase
RIG-I: RNA helicases retinoic acid-inducible gene I
SP: Signal peptidase
SPP: signal peptide peptidase
SUMO-1: Small ubiquitin-like modifier 1
TBSV: Tomato bushy stunt virus
UBC9: Ubiquitin-conjugating enzyme 9
UPS: Ubiquitin proteasome system
